# Nurse uniform wearing practices and associated factors among nurses working in Northwest Ethiopia: a cross-sectional institution based study

**DOI:** 10.1186/s12912-015-0117-3

**Published:** 2015-11-30

**Authors:** Etaferahu Alamaw Desta, Mignote Hailu Gebrie, Berihun Assefa Dachew

**Affiliations:** Addis Ababa Black Lion Hospital, Addis Ababa, Ethiopia; Department of Nursing, College of Medicine and Health Sciences, University of Gondar, Gondar, Ethiopia; Department of Epidemiology and Biostatistics, Institute of Public Heath, College of Medicine and Health Science, University of Gondar, Gondar, Ethiopia

**Keywords:** Nurses, Nursing Uniform, Northwest Ethiopia, Uniform wearing practice

## Abstract

**Background:**

Wearing uniforms help in the formation of professional identity in healthcare. It fosters a strong self image and professional identity which can lead to good confidence and better performance in nursing practice. However, most nurses in Ethiopia are not wearing nursing uniforms and the reasons remain unclear. Therefore, the aim of this research is to assess nurse uniform wearing practices among nurses and factors associated with such practice in hospitals in Northwest Ethiopia.

**Methods:**

A hospital based cross-sectional study was conducted from March to April, 2014 in five hospitals located in Northwest Ethiopia. A total 459 nurses participated in the study. Data was collected using a pre-tested self-administered questionnaire. Descriptive statistics were analyzed in order to characterize the study population. Bivariate and multiple logistic regression models were fitted. Odds ratios with 95 % confidence intervals were computed to identify factors associated with nursing uniform practice.

**Results:**

Nurse uniform wearing practice was found to be 49.2 % of the total sample size. Around 35 % of the respondents that did not implement nurse uniform wearing practices stated that there was no specific uniform for nurses recommended by hospital management. In addition to this, nurse uniform wearing practices were positively associated with being female [AOR = 1.58, 95 % CI (1.02, 2.44)], studying nursing by choice [AOR =3.16, 95 % CI (2.03, 4.92)], and the appeal of nursing uniforms to nurses [AOR = 3.43 95 % CI (1.96, 5.98)].

**Conclusion:**

Nurse uniform wearing practices were not exceptionally prevalent in Northwest Ethiopian hospitals. However, encouraging students to pursue interest-based careers and implementing a nurse uniform wearing policy may have the potential to improve such practices.

**Electronic supplementary material:**

The online version of this article (doi:10.1186/s12912-015-0117-3) contains supplementary material, which is available to authorized users.

## Background

By definition, a uniform can be defined as distinctive attire worn by members of the same body. Nurses in a specific health institution can have a uniform which may differentiate them from other professionals working in the same institution [1]. The first nursing uniforms were influenced by that of the nuns. Before the 19th century, nuns were the primary care takers of patients, essentially serving as the present day nurse. After receiving their nursing training, the nuns acquired the rank of “Sister”. One of the students of Florence Nightingale designed the original uniform for the nursing students. Until the 1940s, this nursing uniform, characterized by its blue color, was fairly standard and underwent minor changes. Later on, responsibility of designing the nursing uniform was given to the hospitals [2]. With this, there is no single uniform recommended for nurses at country level [1].

Uniforms help identify the uniform-wearer, in this case nurses, and their respective roles, namely, to other healthcare professionals and patients. In addition to this, nursing uniforms promote professionalism and cleanliness, while promoting freedom of movement [2, 3]. Wearing a uniform fosters a strong self-image and professional identity which can lead to an improved sense of confidence and ultimately better performance in nursing practice [4].

A study done in School of Nursing, Midwifery and Physiotherapy, at University of Nottingham showed that uniforms play a key role in the delineation of occupational boundaries and the formation of professional identity in healthcare. The result also showed how important uniforms are to their wearers, both in terms of the defense of professional boundaries and status, as well as the construction of professional identity [3].

The same type of uniform may not be preferred by all nurses. A study conducted among patients, nurses, and administrators in College of Nursing, Brigham Young University, reported that uniform comprising of white pants and a stethoscope was preferred among nurses. The white pant uniform with cap, dress with cap, pants suit, and dress with stethoscope scored closely in a second place grouping. The white dress uniform and street clothes with laboratory coat tied for third place. Colored designer scrubs and white pants with colored top scored the lowest [5].

A similar study conducted in a regional medical center compared the responses of healthcare professionals and patients to uniforms similar to those described above.

A majority of respondents agreed that they would prefer to be cared for by the nurse with the dress and stethoscope, while they were least inclined to have the nurse with white pants and colored top care for them [6].

A study conducted on hospitalized Children’s Views of the Good Nurse reported that the majority expected the nurse to wear a uniform that was neat, clean and identified him or her as a hospital employee [7].

In Ethiopia, nurses constitute the majority of health professionals and have the most frequent contact with their patients. Currently, no national and institutional policies or guidelines have been formulated to implement nurse uniform wearing practices. Thus, so far no researches had conducted in Ethiopia about the practice of nurse wearing uniform practices. However, it was observed that a few nurses wore nursing uniforms. The reason for the resistance to wearing nursing uniforms was not well known. This study was conducted to assess nurse uniform wearing practices among nurses and factors associated with such practice in hospitals in Northwest Ethiopia. With this, the study may ultimately help develop policies and guidelines on nurse uniform wearing practices.

## Methods

### Study design

Hospital based cross-sectional study was conducted to assess nurse uniform wearing practices among nurses and factors associated with such practice in hospitals in Northwest Ethiopia from March to April 2014.

### Study setting

The study was conducted in five hospitals in Northwest Ethiopia: the University of Gondar Hospital, Felege Hiwot Hospital, Debretabor Hospital, Metema Hospital and Debark Hospitals.

### Sampling methods

The sample size was calculated using a single population proportion formula with the assumptions of 50 % proportion (p), since there is no similar study, 95 % confidence level (z), and 5 % margin of error (d).

Here is the computation; $\mathrm{n}\kern0.5em =\kern0.5em \frac{z^2\Big(p\left(1-P\right)}{d^2}$

$$ n\kern0.5em =\kern0.5em \frac{1.96^2\Big(0.5\left(1-0.5\right)}{0.05^2}\kern0.5em =\kern0.5em 3.84 $$

With this assumption, the sample size becomes 384. Considering 10 % non response rate the final sample size becomes 422. However, since the total numbers of nurses working in the 5 hospitals are 459, all nurses were included in the study.

### Data collection and analysis

Data was collected using self-administered questionnaire having three parts. The first part contained socio-demographic characteristics of the nurses. The second part of the questionnaire consisted of questions related to nurse uniform wearing practice. The final part of the questionnaire assessed the attitude of the nurses towards the uniform using the five point Likert scale, where 1 indicated strongly disagree and 5 was strongly agree. The questionnaire was pre-tested on 20 nurses working in Woldeya hospital which is found in Amhara Region. After analyzing the pre - test result necessary modifications and corrections were made accordingly before using it in the actual survey. The reliability of the tool was checked using Cronbach’s alpha reliability test with a score of 0.84 (95 % CI 0.801–0.87).

Five nurses and one supervisor were deployed to facilitate data collection. For the purpose of quality assurance, data collectors and facilitators were under close supervision and the collected data was reviewed and checked for completeness, clarity and accuracy on a daily basis prior to data entry.

Data was entered using EPI-INFO version 3.5.3 and exported to SPSS version 20 statistical software for further analysis. Descriptive statistics were carried out to characterize the study population using different variables. Both bivariate and multiple logistic regressions were used to identify associated factors. Crude and adjusted odds ratio with 95 % confidence interval (CI) were calculated to determine the strength and presence of association. Variables with *P*-value ≤ 0.05 were considered as having a significant association with the practice of wearing nurse uniform.

### Ethics statement

Ethical clearance was obtained from the Department of Nursing Ethical Review Committee at the University of Gondar (RfNo: 1073/07/06). A letter of cooperation was written to the hospitals by the Department of Nursing and permission to conduct the research was obtained from each hospital. Written informed consent was also obtained from the study subjects. It was explained that their participation is on a voluntary basis. Personal identifiers were not included in the written questionnaires to ensure participants’ confidentiality.

## Results

Four hundred fifty nine (459) nurses participated in the study. 45.3 percent (208) of these nurses were from Gondar University hospital followed by Felege Hiwot hospital 31.2 percent (143). Of the 459 nurses, 231 respondents (50.3 %) were males. The mean age of the respondents was 28.8 ± 6.1 years. While the minimum age was 20 years, the maximum was 56 years. About 66.5 percent of the respondents are between 20 and 29 years old.

The minimum work experience of the respondents was 1 month and the maximum was 36 years. The mean work experience was 5.5 ± 5.4 years. 39.4 percent of the respondents were from the internal medicine department, 16.6 percent were from surgery/orthopedics, and 16.3 percent from pediatrics. Two hundred nine (45.5 %) of the respondents studying nursing without their preference (Table 1).

**Table 1:** Socio-demographic characteristics of nurses working in Hospitals of Northwest Ethiopia, March 2014 (*n* = 459)

Characteristics	Number	Percent
Hospitals (Work place)		
University of Gondar Hospital	208	45.3
Felege Hiwot Hospital	143	31.2
Debre Tabor Hospital	66	14.4
Debark Hospital	23	5.0
Metema Hospital	19	4.1
Sex		
Male	231	50.3
Female	228	49.7
Age		
20–24	110	24.0
25–29	195	42.5
30–34	81	17.6
35–39	35	7.6
40–56	38	8.3
Religion		
Orthodox Christian	390	85. 0
Protestant	37	8.1
Muslim	29	6.3
Others*	3	0.7
Qualification		
Diploma	157	34.2
Degree	290	63.2
Master	12	2.6
Work experience as a nurse		
<5 years	276	60.1
5–9 years	115	25.1
10–14 years	29	6.3
15–19 years	24	5.2
20–36 years	15	3.3

* refers to Catholic and Adventist

### Nursing uniform wearing practice

49.2 percent of the surveyed nurses (226 nurses) wore uniforms. Debark hospital had the highest percentage (65.2 %) of nurses wearing uniforms, while Gondar University Hospital had the lowest (38.0 %). When considering the gender of the surveyed nurses, it was observed that uniform wearing practice was higher among females 137 (60.1 %) than males 89 (38.9 %). 35.2 percent or 88 of nurses did not wear uniforms because there was no specific uniform that was recommended by the hospital management. Whereas 24.3 percent, or 55 nurses, chose not to wear the recommended uniforms by choice even though the hospital management recommended the type of uniform which shall be worn by nurses (Table 2).

**Table 2:** Reasons for not wearing nurse uniform in hospitals of North West Ethiopia, March 2014 (*n* = 233)

Reasons	Number	Percent
There is no specific uniform for nurses which is	82	35.2
recommended by the hospital management		
I don’t like to wear a nurse uniform	55	23.6
It doesn’t matter whether I wear a nurse uniform or not	33	14.2
I don’t want to be recognized as a nurse	39	16.7
More than one reason	9	3.9
Other^a^	5	2.1

^a^Others include (The weather condition is not comfortable, it is time taking, not important)

### Factors associated with nurse uniform wearing practice

In Bivariate analysis, being a female, having work experience as a nurse, freely choosing to study nursing, and personal preference for the nursing uniform were significantly associated with nurses uniform wearing practice. However, only gender (being female) [AOR = 1.58, 95 % CI, (1.02, 2.44)], studying nursing by preference [AOR =3.16, 95 % CI (2.03, 4.92)] and the attitude of nurses towards the nursing uniform [AOR = 3.43 95 % CI (1.96, 5.98)] showed significant association with nurse uniform wearing practice during multivariate analysis (Table 3).

**Table 3:** Factors associated with nursing uniform wearing practice in hospitals of Northwest Ethiopia, March 2014 (*n* = 459)

Variables	Uniform wearing practice		COR (95 % CI)	AOR (95 % CI)
	Yes	No		
Sex				
Male	89(38.5 %)	142(61.5 %)	1	1
Female	137(60.1 %)	91 (39.9 %)	2.40(1.65,3.49)	1.58(1.02,2.44)*
Qualification				
Diploma	95(60.5 %)	62(39.5 %)	1	1
Degree and above	131(43.4 %)	171(56.6 %)	0.5(0.34,0.74)	0.66(0.39,1.11)
Work experience				
<5 years	132(47.8 %)	144(52.2 %)	1	1
5-9 years	61(53.0 %)	54(47.0 %)	1.3(1.06,1.75)	0.71(0.31,1.62)
10-36 years	35(48.5 %)	35(51.5 %)	0.84(0.46,1.52)	0.83(0.37,1.89)
Studding nursing by preference				
No	63(30.1 %)	146(69.9 %)	1	1
Yes	163(65.2 %)	87(34.8 %)	4.34(2.93,6.44)	3.16(2.03,4.92)*
Attitude towards nursing uniform				
Unfavorable	25(20.8 %)	95(79.2 %)	1	1
Favorable	201(59.3 %)	138(40.7 %)	5.54(3.39,9.04)	3.43(1.96,5.98)*

*Statistically significant at *P*-value ≤ 0.05, *COR* crude odds ratio, *AOR* Adjusted odds ratio

## Discussion

Nurses constitute the majority of health professionals in the hospitals and have the most frequent contact with their patients compared to other health professionals. Wearing a uniform conveys a message about the responsibilities of an individual, particularly with respect to his or her job. Such a message has meaning for the wearer, the organization, and the public in general. Additionally, wearing the uniform can foster a strong self image and professional identity which can lead to greater confidence and better performance by the wearer [2–4].

While nurse uniform wearing practices are advantageous, only 49.2 % of the nurses reported to wearing a uniform. This indicates that more than half of the nurses are not wearing a uniform. Even though there were no previous studies to compare the results with, it was clear that large proportions of the nurses in the selected hospitals are not wearing nurse uniforms. In this study, when inquired about why they did not wear uniforms, 35.2 % of the surveyed nurses reported that there was no specific uniform for nurses which was recommended by the hospital management. Whereas, 24.3 % said I don’t like to wear a nurse uniform, even though the hospital management has recommended the type of uniform which shall be worn by nurses. These two reasons seem contradictory. On one hand it seems that there is no standard uniform recommended by the hospital management. On the other hand, a significant number of the nurses reported that they didn’t want to wear nurse uniform, even though the specific type of uniform was recommended by the hospital management. The lack of national and institutional policy and guidelines in Ethiopia regarding nurse uniform may be the reason for such confusion. It is found in literature that different countries have their own national policies and guidelines to guide the wearing of uniform by the nurses [8–12].

Wearing uniform is believed to foster a strong self image and professional identity [2–4]. About 74 % of the surveyed nurses are in favor of wearing nursing uniforms. Wearing a nursing uniform also allows nurses to be easily identified by supervisors, coworkers, and patients.

Being a female, having work experience as a nurse, freely choosing to study nursing, and personal preference for the nursing uniform were significantly associated with nurses uniform wearing practice. According to the study, females wear the nursing uniform 1.58 times more often than males. The first nursing uniforms were inspired by that of the nuns, the initial care-takers for patients. As a result, as nurses began caring for the patients, they adopted the behavior of the nuns and acquired a rank of “Sister” [2]. This might have influenced females to have a more favorable attitude towards nurse uniform wearing practices than males. Moreover, the nurses who had a favorable attitude towards wearing the uniform were 3.43 times more likely to have a good wear uniforms than their unwilling counterparts.

It was also found that those nurses who studied nursing by their preference were 3.16 times more likely to wear nursing uniforms than those who studied nursing under some form of obligation. In Ethiopia, since students are assigned by the Ministry of Education [13] to pursue tertiary education, those who fail to score higher marks may not get the opportunity to study a profession which is their first choice. In such circumstances, students may be assigned to study a profession which is their second or lower choice. Hence, such results are not surprising.

One limitation of this study is that its primary focus was on individual factors, rather than the health system as a whole. In addition to this, the data was collected by a self-reported questionnaire, which may have led to a reporting bias. Furthermore, the study is not triangulated with qualitative methods.

## Conclusion

Nurse uniform wearing practice was found to be low. Strengthening uniform wearing policy and assigning students to careers based on their interest would improve nurse uniform wearing practice.

## Additional file

Additional file 1:
**English version questionnaire.** (PDF 335 kb)
