# Supplementary material for: Nurse uniform wearing practices and associated factors among nurses working in Northwest Ethiopia: a cross-sectional institution based study
Source: BMC Nurs. 2015 Nov 30;14:65. doi: 10.1186/s12912-015-0117-3 (PMC4663730; doi:10.1186/s12912-015-0117-3)
Supplement: Additional file 1: — English version questionnaire. (PDF 335 kb) [file 12912_2015_117_MOESM1_ESM.pdf]

English version questionnaire

Title of research: Nurse Uniform Wearing Practices and Associated Factors among Nurses Working in Northwest Ethiopia: A cross-sectional Institution Based Study.

Consent sheet

Dear participants;

Greetings;

This study is proposed to assess Nurse Uniform Wearing Practices and Associated Factors among Nurses Working in Northwest Ethiopia. This questionnaire is divided into three parts: Question related to socio-demographic characteristics, Nurse uniform wearing practices and attitude of the nurses towards the uniform. There is no harm in participating in this research. Your names and other personal identifies will not be written on the questionnaire. We assure you that all the information you gave will be confidential. Participation in the study is on voluntary basis, you have a full write to participate or not to participate in the study. However, the findings of this study are believed to contribute the development of policies and guidelines related to nurse uniforms. Hence, you are kindly requested to provide genuine answer for the questions included in the questionnaire below. If you need any clarification please do not hesitate to communicate facilitators. For further questions regarding to the study feel free to communicate Mr Berihun Assefa (+251911542348) or Sr. Etaferahu Alamaw (+251939542235).

Thank you for your cooperation;

Are you volunteer to participate in the study

1) Yes ☐
2) No ☐

Signature______________

Part I: Socio- demographic characteristics

S.No.	Questions	Choices/Answers
**101**	Name of hospital you are currently working	1. University of Gondar Hospital<br>2. Felegehiwot Hospital<br>3. Debretabor Hospital<br>4. Metema Hospital<br>5. Debark Hospital
**102**	Sex?	1. Female<br>2. Male
**103**	Age in Years	____________years
**104**	Religion	1. Orthodox Christian<br>2. Muslim<br>3. Protestant<br>4. Catholic<br>5. Other (specify)______
**105**	Qualification	1. Diploma<br>2. Degree<br>3. Masters
**106**	Work experience as a nurse:	_____Years _____ months
**107**	Have you studied nursing by preference	1. Yes, I studied nursing by preference<br>2. No, Circumstances forced me to study nursing

Part II: Questions related to nurse uniform wearing practices

S.No.	Questions	Possible responses
**201**	Are you currently wearing nurse uniform while you providing health service in the hospital	A) Yes<br>B) No<br>**If your answer is 'No' please go to question number 3**
**202**	If your answer to question number 1 is 'Yes',<br>How frequently you wear the nurse uniform?	a) Always whenever I am providing health service in the hospital<br>b) Most of the time<br>c) Some times
**203**	If your answer to question number 9 is "No", why aren't you wearing a nurse uniform?<br>_	A) There is no specific uniform for nurses which is recommended by the hospital management<br>B) I don't like to wear a nurse uniform even though the hospital management has recommended the type of uniform which shall be worn by nurses<br>C) It doesn't matter whether I wear a nurse uniform or not<br>D) I don't want to be recognized as a nurse<br>E) Other specify:<br>--<br>--

Part III. Question related to the attitude of the nurses towards the nurse uniform

Directions: This part of the questionnaire assesses your opinion about your feelings on nurse uniform. Indicate your opinion about each statement that would apply for you by circling:

**Attitude statement**	**Strongly agree (5)**	**Agree (4)**	**Neutral (3)**	**Disagree (2)**	**Strongly disagree (1)**
It should be easy to identify nurses as nurses	5	4	3	2	1
It is important to be able to recognize the nurse in charge by the cloths she/he wears	5	4	3	2	1
Wearing nurse uniform reflects respect for the profession	5	4	3	2	1
All health professionals (doctors, nurses, laboratory technologists etc) should **not** wear the same type of uniform	5	4	3	2	1
Nurses should wear the same kind of clothing	5	4	3	2	1
Nurses should feel proud when they wear nurse uniform	5	4	3	2	1
I am willing to wear nurse uniform in the future	5	4	3	2	1
I appreciate those nurses who wear nurse uniform	5	4	3	2	1
I was comfortable in wearing nurse uniform when I was nursing student	5	4	3	2	1

This is the end of the questionnaire! Thank you for your participation!
